# First Report of Ciguatoxins in Two Starfish Species: *Ophidiaster ophidianus* and *Marthasterias glacialis*

**DOI:** 10.3390/toxins7093740

**Published:** 2015-09-21

**Authors:** Marisa Silva, Inés Rodriguez, Aldo Barreiro, Manfred Kaufmann, Ana Isabel Neto, Meryem Hassouani, Brahim Sabour, Amparo Alfonso, Luis M. Botana, Vitor Vasconcelos

**Affiliations:** 1 Department of Biology, Faculty of Sciences, University of Porto, Rua do Campo Alegre, Porto 4619-007, Portugal; E-Mails: marisasilva17@gmail.com (M.S.); aldo.barreiro@gmail.com (A.B.); 2 Interdisciplinary Center of Marine and Environmental Research—CIMAR/CIIMAR, University of Porto, Rua dos Bragas, 289, Porto 4050-123, Portugal; E-Mails: mkaufmann@ciimar.up.pt (M.K.); aneto@uac.pt (A.I.N.); 3 Department of Pharmacology, Faculty of Veterinary, University of Santiago of Compostela, Lugo 27002, Spain; E-Mails: ines.rodriguez.filgueiras@usc.es (I.R.); amparo.alfonso@usc.es (A.A.); Luis.Botana@usc.es (L.M.B.); 4 Centre of Life Sciences, University of Madeira, Marine Biology Station of Funchal, Funchal 9000-107, Portugal; 5 Center of Interdisciplinary Marine and Environmental Research of Madeira—CIIMAR-Madeira, Edif. Madeira Tecnopolo, Caminho da Penteada, Funchal 9020-105, Portugal; 6 Department of Marine Biology, University of Azores, Ponta Delgada 9501-801, Portugal; 7 Phycology Research Unit—Biotechnology, Ecosystems Ecology and Valorization Laboratory. Faculty of Sciences El Jadida, University Chouaib Doukkali, El Jadida BP20, Morocco; E-Mails: hassouani@hotmail.com (M.H.); sabour.b@ucd.ac.ma (B.S.)

**Keywords:** ciguatera, new vectors, Madeira Island, São Miguel Island, Morocco

## Abstract

Ciguatera fish poisoning (CFP) is a syndrome caused by the ingestion of fish contaminated with Ciguatoxins (CTXs). These phycotoxins are produced mainly by dinoflagellates that belong to the genus *Gambierdiscus* that are transformed in more toxic forms in predatory fish guts, and are more present in the Indo-Pacific and Caribbean areas. It is estimated that CFP causes per year more than 10,000 intoxications worldwide. With the rise of water temperature and anthropogenic intervention, it is important to study the prevalence of CFP in more temperate waters. Through inter- and subtidal sampling, 22 species of organisms were collected, in Madeira and Azores archipelagos and in the northwestern Moroccan coast, during September of 2012 and June and July of 2013. A total of 94 samples of 22 different species of bivalves, gastropods, echinoderms and crustaceans where analyzed by Ultra Performance Liquid Chromatography-Mass Spectometry-Ion Trap-Time of Flight (UPLC-MS-IT-TOF) and Ultra Performance Chromatography- Mass Spectrometry (UPLC-MS). Our main aim was to detect new vectors and ascertain if there were some geographical differences. We detected for the first time putative CTXs in echinoderms, in two starfish species—*M. glacialis* and *O. ophidianus*. We detected differences regarding uptake values by organisms and geographical location. Toxin amounts were significant, showing the importance and the need for continuity of these studies to gain more knowledge about the prevalence of these toxins, in order to better access human health risk. In addition, we suggest monitoring of these toxins should be extended to other vectors, starfish being a good alternative for protecting and accessing human health risk.

## 1. Introduction

Ciguatoxins (CTXs) are lipophilic compounds that result from the biotransformation in finfish of precursor gambiertoxins produced by dinoflagellates from the genus *Gambierdiscus* [1,2,3]. Geographically, CTXs are typical for the tropical and sub-tropical regions of the Atlantic (C-CTX), Indian (I-CTX) and Pacific (P-CTX) Oceans [1,4,5,6,7,8]. Both the structure and potency of CTXs appear to be geographically determined, in resemblance to Maitoxin that shares the same producer, and to date more than 30 CTX analogues have been described [3,4,5,7,9,10,11,12,13].

**Figure 1:**
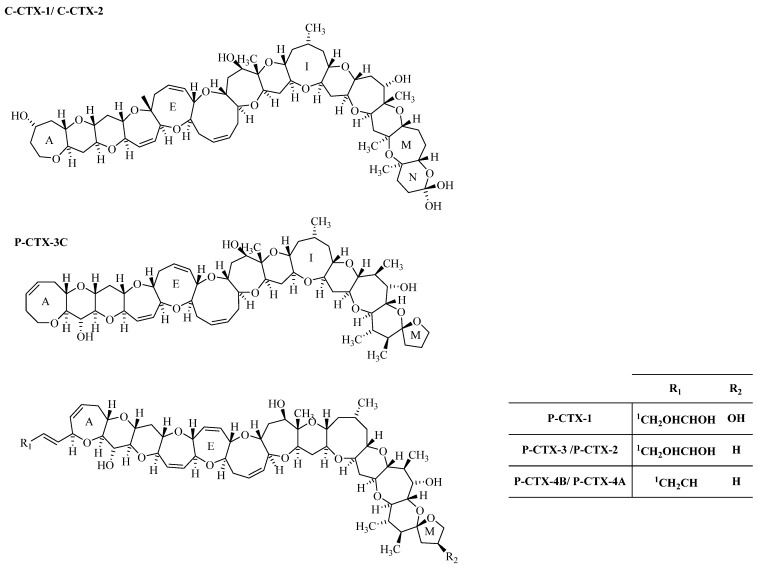
Structures Caribbean (C) and Pacific (P) CTX-group toxin. The epimers, P-CTX-2 (52-epi P-CTX-3), P-CTX-4A (52-epi P-CTX-4B) and C-CTX-2 (56-epi C-CTX-1) are indicated in parenthesis.

CTXs are polyethers with rigid structure, formed by rings 13–14 fused by ether linkages (Figure 1) [2]. These potent biotoxins are odorless, tasteless, heat resistant and lipid soluble, so they are not destroyed by cooking processes [7]. Regarding action mechanism, CTXs bind to non-selective, non-voltage activated ion channels, causing their opening, leading to the increase of intracellular calcium levels to toxic concentrations [14]. In terms of symptomatology, during the acute period in the first 24 h gastrointestinal problems such as diarrhea, nausea, abdominal pain and vomiting appear. In addition, cardiovascular complications can occur. Neurologic symptoms that can emerge from within a few hours to two weeks after exposure, like paresthesias, dysesthesias, and hyperesthesias [15].

Ciguatera fish poisoning (CFP) is the most common type of intoxication syndrome, even in non-endemic areas, due to the large quantity of fish exports, and this makes it a worldwide concern [16]. The most common route of intoxication is through ingestion of contaminated fish, being estimated 10,000 to 50,000 intoxications per year [4,17]. Recent reports document the presence of CTXs as far north as the Mediterranean [18]. In addition, high abundance of *Gambierdiscus* cells are not visible as blooms, as in other harmful algal bloom species, and this hinders the process of monitoring and managing of these blooms [19].

The common vectors for these phycotoxins are finfish, some mollusks (e.g., turban snail, *Lunella cinerea*, and in giant clams, *Tridacna gigas*), achieving higher concentrations in top predatory fish like groupers (Family: *Serranidae*), barracuda (Family: *Sphyraenidae*) and snapper (Family: *Lutjanidae*) [20]. Regarding the regulatory status in the European Union (EU), EFSA proposes the use of toxicity equivalency factors (TEFs), based on their acute intraperitoneal LD_50_ in mice, determining eleven values: P-CTX-1 = 1, P-CTX-2 = 0.3, P-CTX-3 = 0.3, C-CTX-2 = 0.3, P-CTX-3C = 0.2, 2,3-dihydroxy PCTX-3C = 0.1, 51-hydroxy P-CTX-3C = 1, P-CTX-4A = 0.1, C-CTX-1 = 0.1 and P-CTX-4B = 0.05. In the same scientific opinion on CTXs, EFSA claims that a concentration of 0.01 μg P-CTX-1 equivalents/kg of fish as expectable not to exert effects in sensitive individuals. In addition, due to very few reported cases for CTX occurrences European markets no limits nor ARfD (acute reference dosage) have been established until now, nor advisement regarding analytical methodologies to use [1]. Nevertheless, Commission Regulation (EC) nr 854/2004stated mandates that checks have to be made to ensure that fishery products containing CTX are forbidden to enter the market [21]. Other countries like the United States of America, have safety levels (<0.1 μg/kg C-CTX-1 equivalents and <0.01 μg/kg P-CTX-1 equivalents), other countries opted for more radical measures, like Japan that banned the importation of some fish species reported as CFP vectors (e.g., barracuda) [1,22].

The aim of our work was to search for new vectors, to better access the human health risk. We screened the Madeira Island (Madeira archipelago), São Miguel Island (Azores archipelago), and the Moroccan coast by scuba diving expeditions and intertidal harvesting of 22 edible and inedible species. The inedible species were collected due to their importance in the food chain. We hope that our work contributes to the development and establishment of monitoring procedures as well as legislation in the EU to better protect consumers and public health.

## 2. Results and Discussion

**Figure 2:**
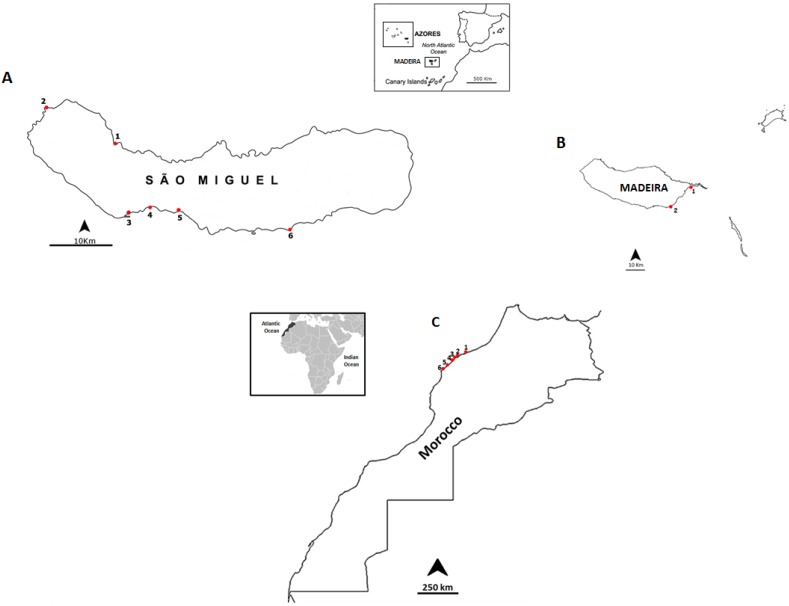
Location of the sampling points: (**A**) São Miguel island coast, Azores archipelago: 1, Cruzeiro; 2, Mosteiros; 3, Étar; 4, São Roque; 5, Lagoa; and 6, Caloura. (**B**) Madeira island coast: 1, Reis Magos and 2, Caniçal. (**C**) Northwestern Moroccan coast: 1, Casablanca Corniche; 2, El Jadida Haras; 3, El Jadida Sâada; 4, Sidi Bouzid; 5, Mrizika; and 6, Oualidia.

**Figure 3:**
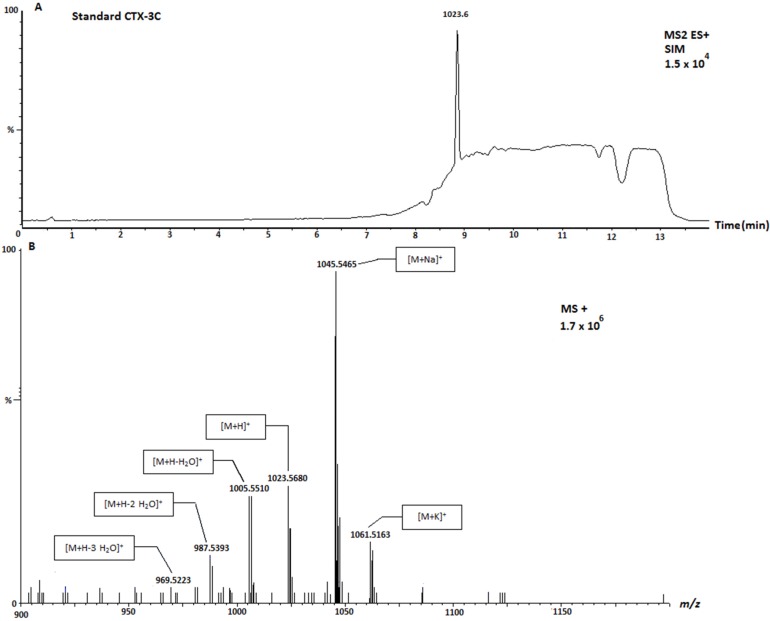
Selected Ion Monitoring (SIM) chromatogram (**A**) and mass spectrum (**B**) of standard CTX-3C. SIM obtained by UPLC-MS/MS and spectrum obtained by UPLC-MS-IT-TOF.

**Figure 4:**
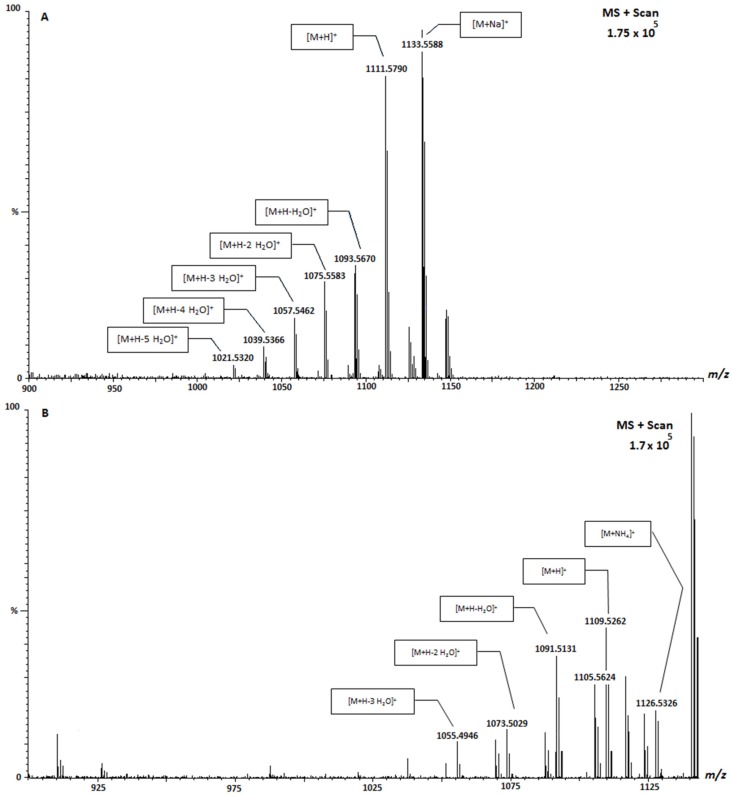

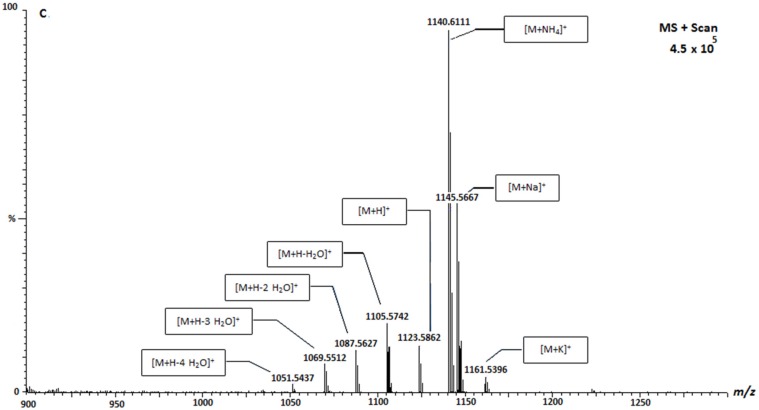
MS2 spectrum of CTX analogue at *m*/*z* 1111.5 [M+H]^+^ (**A**); CTX analogue at *m*/*z* 1109.5 [M+H]^+^ (**B**); and CTX analogue *m*/*z* 1123.5 [M+H]^+^ (**C**). Spectra obtained by Ultra Performance Liquid Chromatography-Mass Spectometry-Ion Trap-Time of Flight UPLC-MS-IT-TOF.

In this work, a total of 94 samples were analyzed for CTX plus its analogues from 14 sampling points distributed in three different locations: Madeira Island (Figure 2), São Miguel Island (Figure 3) and along the northwestern Moroccan Coast (Figure 4). Twenty-two species of benthic organisms, including bivalves (mussels), gastropods (sea-snails, sea-slugs and limpets), echinoderms (starfishes, sea-urchins and sea-cucumbers) and crustaceans (barnacles) were collected. The decision to sample these particular species was linked to the fact we wanted to screen edible and commercially important species and inedible species for their importance in the trophic chain. The number of samples collected and average number of specimens needed to set a pooled sample are detailed (Table 1).

**Table 1:** Species sampled and their trophic level, average number of specimens comprising a pooled sample (AvNr) and number of samples collected (NrP Samples) on Madeira Island in September 2012, São Miguel Island, Azores, in June 2013 and Morocco in July 2013 and their edibility. Availability of animals is dependent on their geographical distribution and ecology.

Species	Trophic Level	Sampling Site(s)	NrP Samples	AvNr	Edible	Ref.
*Aplysia depilans*	Grazer	Morocco	3	1	No	[23]
*Arbacia lixula*	Grazer	Madeira/Azores/Morocco	9	4	No	[24]
*Charonia lampas*	3rd level predator	Madeira/Morocco	3	1	Yes	[25]
*Cerithium vulgatum*	Grazer	Morocco	1	40	Yes	[26]
*Diadema africanum*	Grazer	Madeira	2	1	No	[27]
*Echinaster sepositus*	2nd level predator	Madeira	1	3	No	[28]
*Gibbula umbilicalis*	Grazer	Morocco	3	100	Yes	[29]
*Holothuria(Platyperona)sanctori*	Deposit feeder	Morocco	4	1	Yes	[30,31]
*Marthasterias glacialis*	2nd level predator	Madeira/Azores/Morocco	8	1	No	[32]
*Monodonta lineata*	Grazer	Morocco	5	86	Yes	[29]
*Mytilus* spp.	Filter feeder	Morocco	4	30	Yes	[33]
*Onchidella celtica*	Grazer	Morocco	1	50	No	[34]
*Ophidiaster ophidianus*	Detritivorous	Madeira/Azores	5	1	No	[28]
*Pattela aspera*	Grazer	Madeira	2	15	Yes	[32]
*Patella* spp.	Grazer	Morocco	4	12	Yes	[32]
*Pattela candei*	Grazer	Azores	3	10	Yes	[32]
*Paracentrotus lividus*	Grazer	Madeira/Azores/Morocco	7	1	Yes	[35]
*Pollicipes pollicipes*	Filter feeder	Morocco	3	35	Yes	[32]
*Sphaerechinus granularis*	Grazer	Azores	4	1	Yes	[36]
*Umbraculum umbraculum*	Grazer	Madeira	1	1	No	[37]
*Stramonita haemostoma*	2nd level predator	Madeira/Azores/Morocco	5	15	No	[38]

After sample extraction, the CTXs profile was determined in each sample by Ultra Performance Liquid Chromatography- Mass Spectometry (UPLC-MS) method. Initially, samples were screened by UPLC-MS in Selected Ion Monitoring (SIM) mode for 20 CTXs analogues described in the literature (Table 2) and in seven samples some suspicious peak was detected, all of them from starfish.

**Table 2:** Screened CTXs UPLC-MS in Positive Mode.

Compound Name	Mass	Polarity
I-CTX-3 and I-CTX-4	1157.6	Positive
Unknown CTX	1143.6	Positive
Caribean-CTX	1141.7	Positive
C-CTX-1127	1127.6	Positive
CTX-1B	1111.6	Positive
54-deoxy-CTX-1B 52-epi-54-deoxy-CTX-1B	1095.6	Positive
M-CTX-4A and M-CTX-4B	1079.6	Positive
CTX-4A and CTX-4B	1061.6	Positive
MTX small	1060	Positive
2,3-OH-CTX-3C M-CTX-3C methylacetal	1055.6	Positive
2-OH-CTX-3C and M-CTX-3C	1041.6	Positive
Analogs CTX	1040.6	Positive
51-OH-CTX-3C	1039.6	Positive
49-epo-CTX-3C and CTX-3C	1023.6	Positive
Unknown CTX	1159.6	Positive

Afterwards, the scan of each sample was carefully analyzed, checking the characteristic mass pattern fragmentation of CTX; that is, the formation of sodium and ammonium adducts and losses of water. In this sense, Figure 3 shows the SIM and the spectrum of CTX-3C standard. The mass spectrum from Figure 3B showed the ion CTX3C [M + H]^+^ *m*/*z* 1023.5, and the water losses *m*/*z* 1005.5 associated with [M + H − H_2_O]^+^ and *m*/*z* 987.5 due to second water lost [M + H − 2H_2_O]^+^. In addition, the sodium adduct [M + Na]^+^ *m*/*z* 1045.7 is also detected.

Through this analysis, three CTX analogues were detected. From SIM data of samples, *m*/*z* 1111.5 was found and from scan mass mode (*m*/*z* 800–1300) of samples, two molecules with *m*/*z* 1109.5 and *m*/*z* 1123.5 were found. The mass spectrum of these molecules (Figure 4) shows the typical fragmentation pattern of CTX-like compounds, with several losses of water and adducts of ammonium and sodium. The mass spectrum in Figure 4A shows two intense masses, one ([M + H]^+^) at *m*/*z* 1111.5 and other ([M + Na]^+^) at *m*/*z* 1133.5. Moreover, five losses of water were observed while the ammonium adduct did not appear, probably due to its low intensity. Therefore CTX-1B, *m*/*z* 1111.5, could be proposed, although not confirmed due to the absence of a standard. The mass spectrum in Figure 4B shows an intense mass ([M + NH_4_]^+^) at *m*/*z* 1116.6 associated with ammonium adduct from *m*/*z* 1109.5. The other three masses shown in the spectrum correspond to one, two and three losses of water at *m*/*z* 1099.6 and 1073.5 and 1055.5, respectively, from *m*/*z* 1109.5 molecule. The mass spectrum in Figure 4C shows an ion at [M + H]^+^ *m*/*z* 1123.5 and two intense masses, [M + NH_4_]^+^ at *m*/*z* 1140.5 and [M + Na]^+^ at *m*/*z* 1145.5. In addition, the four losses of water and the potassium adduct of *m*/*z* 1123.5 were also detected in the spectrum.

Once the presence of CTX analogues was identified, the toxin amount was quantified (Table 3). In order to calculate concentrations of CTX analogues, it was assumed that related analogues would give a similar response to that of CTX-3C, because of this, and since there is an absence of correspondence with EFSAs, TEFs results will be expressed in μg CTX-3C equivalents/Kg. Therefore, the calibration curve was done with CTX-3C standard. The quantification of these toxins was carried out using SIM acquisition in positive mode. For CTX-3C standard, a good seven-point calibration curve with range between 18.75–600 ng/mL was obtained (R2 = 0.999). The limit of detection (LOD) was 1.125 µg/Kg and the limit of quantification (LOQ) was 3.75 µg/Kg.

**Table 3:** Presumed CTX detected in UPLC-MS (FW-Fresh weight).

Sample	Location	Species	1111 (μg CTX-3C Equivalents/KgFw)	1123 (μg CTX-3C Equivalents/KgFw)	1109 (μg CTX-3C Equivalents/KgFw)
341 #1	Madeira	*O. ophidianus*	21.55	7.24	
341 #2	Madeira	*O. ophidianus*	46.49	29.80	
411 #2	Azores	*M. glacialis*	<LOQ	<LOQ	
412	Azores	*O. ophidianus*	19.73		
424	Azores	*O. ophidianus*	124.04	30.90	
426 #1	Azores	*M. glacialis*	7.92		
435	Azores	*O. ophidianus*	58.78		4.40

Only 7.45% of the 94 samples analyzed were quantifiable for CTX. All of the measurable samples were one of two starfish species, *O. ophidianus* and *M. glacialis*, from Portuguese territory. This is the first report of CTX in echinoderms. Average concentrations detected ranged from 4.40 μg CTX-3C equivalents/Kg fresh weigh (fw), to 124.04 μg CTX-3C equivalents/Kg fw in *O. ophidianus*, if it were possible to apply EFSAs, TEFs both would be above the U.S. American safety level (<0.1 μg/kg C-CTX-1 equivalents and <0.01 μg/kg P-CTX-1 equivalents) [22]. Regarding statistics, the first step of the gamma hurdle model was a GLZ performed with the data of presence/absence of CTX with Binomial distribution of the error. The results of the model’s analysis of deviance are shown in Table 4, as well as the coefficients rescaled to a logistic probability (0,1). Sampling site had no significant effect. Among sampling sites, the coefficients showed the lowest probability of occurrence in Morocco, and the highest in Madeira. The second part of the model, which considers the variation in CTX concentration, analyzed with a Gamma distribution of the error among those samples showing positive results, did not show significance of sampling site as factor either, although its *p* value was closer to significance (Table 4). Looking at model coefficients, it is clear that the largest concentrations found occurred in Azores Islands, and lowest in Morocco (zero). This differs from the probability results of the presence/absence data.

The analysis performed with starfish species as factor (Table 5) showed significant differences in both the presence/absence and the analysis of the positives (differences in putative CTX concentrations), conclusively, *O. ophidianus* had both higher probability of showing a positive result, and significantly higher amounts of toxin per body mass. This can be explained by to their distinct feeding habits, since *O. ophidianus* is a detritivorous acquiring CTX from the sediment bed, against the predatory ones from *M. glacialis*. In addition, Llewellyn (2010) suggested that the rise of water temperature, derived by climate change, can increase the incidence of CFP in Papua New Guinea from 35–70 per thousand people in 1990 to 160–430 per thousand people in 2050 [39]. In addition, it is known that the rise of water temperature can influence the growth rate of the producers [40], toxin production as well as the uptake rate of the vectors [41,42], and that could be the main reason of the differences between sampling sites, though Morocco and Madeira are at the same latitude. Nevertheless, the main species that we detected quantifiable amounts of CTX is absent in Morocco. Since Madeira and Azores archipelagos present oligotrophic waters, making these ecosystems poor in bivalves, the red starfish (*O. ophidianus*) presents a possible alternative as key species for CTX monitoring. We have to interpret the results from our models carefully, since the actual number of data is low. This may affect the non-significance of the models from sampling sites (Table 4) where the differences found might become significant by simply adding more data. However, the models using species as factor (Table 5) reveal a strong pattern that is not affected by the small number of data.

**Table 4:** Results of the gamma hurdle model for CTX occurrence with sampling site as factor.

Analysis of Deviance				
Model	Factor	χ^2^	*df*	*p*
**Binomial Error**	Site	2.9	2	0.23
	Rescaled model coefficients for Site: Intercept (Azores) = 0.36; Madeira = 0.78; Morocco = 4 × 10^−8^			
**Gamma Error**	Site	5.5	2	0.06
	Rescaled model coefficients for Site: Intercept (Azores) = 229; Madeira = 1.39; Morocco = 0.004			

**Table 5:** Results of the gamma hurdle model for CTX occurrence with starfish species as factor.

Analysis of Deviance				
Model	Factor	χ^2^	*df*	*p*
**Binomial Error**	Species	8.5	1	<0.01
	Rescaled model coefficients: Intercept (*M. glacialis*) = 0.11; *O. ophidianus* = 0.98			
**Gamma Error**	Organism	9.7	1	<0.01
	Rescaled model coefficients: Intercept (*M. glacialis*) = 9.4; *O. ophidianus* = 57.7			

Our results add new information to this topic, we can say, to the best of our knowledge, that this is the first report of putative CTX in starfish. It is also noteworthy that significant amounts of this group of toxins were detected at the bottom of the trophic chain. We hope this contribute towards the establishment of legislation, as well as the promotion of the monitoring of these toxins in the EU.

## 3. Experimental Section

### 3.1. Selected Species and Sampling Sites

The coasts of the Portuguese islands Madeira (Madeira archipelago), São Miguel (Azores archipelago) and the northwestern coast of Morocco, were surveyed for non-traditional vector species for Ciguatoxins. Several edible and non-edible species were selected (*n* = 22) to search for potential new vectors and also the prevalence of the screened biotoxins in the food web: gastropods (*Aplysia depilans*, *Cerithium vulgatum*, *Charonia lampas*, *Gibbula umbilicalis*, *Monodonta lineata*, *Onchidella celtica*, *Patella tenuis tenuis*, *Patella aspera*, *Patella candei*, *Patella* spp., *Stramonita haemostoma*, *Umbraculum umbraculum*), crustaceans (*Pollicipes pollicipes*), bivalves (*Mytilus* spp.), starfish (*Echinaster sepositus*, *Marthasterias glacialis*, *Ophidiaster ophidianus*), sea-cucumber (*Holothuria (Platyperona) sanctori*), sea-urchins (*Arbacia lixula*, *Diadema africanum*, *Paracentrotus lividus*, *Sphaerechinus granularis*). Benthic organisms were harvested from intertidal areas during low tide and by scuba diving expeditions: the Madeira Island was surveyed in September 2012, and São Miguel Island, Azores, and the Moroccan coast were sampled in June and July 2013, respectively. Sampling sites are displayed in Table 6.

Two samples of *Patella tenuis tenuis* and *P. aspera* were purchased in local markets in Madeira, being caught in the northern coast of the island (32°51ʹ17.02ʹʹ N; 17°01ʹ54.02ʹʹ W). Organisms were transported to the laboratory in refrigerated containers. Samples were frozen at −20 °C, if they were not processed immediately.

**Table 6:** Sampling Sites and respective geographical coordinates, surveyed during September of 2012 and June and July of 2013.

Date	Location	Sampling Site	Geographic Coordinates
September 2012	Madeira Island	Reis Magos	32°39ʹ16.21ʹʹ N; 16°49ʹ05.29ʹʹ W
		Caniçal	32°44ʹ20.08ʹʹ N; 16°44ʹ17.55ʹʹ W
June 2013	São Miguel Island	Cruzeiro	37°50ʹ31.19ʹʹ N; 25° 41ʹ33.61ʹʹ W
		Étar	37°44ʹ19.31ʹʹ N; 25°39ʹ38.84ʹʹ W
		São Roque	37°45ʹ15.35ʹʹ N; 25°38ʹ31.60ʹʹ W
		Mosteiros	37°53ʹ25.57ʹʹ N; 25°49ʹ14.72ʹʹ W
		Lagoa	37°44ʹ42.38ʹʹ N; 25°19ʹ.47ʹʹ W
		Caloura	37°42ʹ49.34ʹʹ N; 25°29ʹ54.54ʹʹ W
July 2013	Morocco Coast	Casablanca corniche	33°36ʹ01.2ʹʹ N; 7°39ʹ57.5ʹʹ W
		El Jadida Haras	33°14ʹ42.0ʹʹ N; 8°28ʹ37.5ʹʹ W
		El Jadida Sâada	33°14ʹ42.4ʹʹ N; 8°32ʹ26.9ʹʹ W
		Sidi Bouzid	33°13ʹ57.1ʹʹ N; 8°33ʹ20.9ʹʹ W
		Mrizika	32°57ʹ21.8ʹʹ N; 8°46ʹ53.2ʹʹ W
		Oualidia	32°43ʹ55.8ʹʹ N; 9°02ʹ57.6ʹʹ W

### 3.2. Reagents

Acetonitrile and methanol were supplied by Panreac (Barcelona, Spain). All solvents employed in this work were high performance liquid chromatography or analytical grade and the water was distilled and passed through a water purification system (Milli-Q, Millipore, Madrid, Spain). Formic acid was purchased from Merck (Darmstadt, Germany). Ammonium formate was from Fluka (Sigma-Aldrich, Madrid, Spain).

A synthetic standard of CTX-3C was provided by Dr. Masahiro Hirama. The methodology applied for the synthesis was described for the first time in 2001 and improved in 2004 [43,44].

### 3.3. Sample Extraction

**Figure 5:**
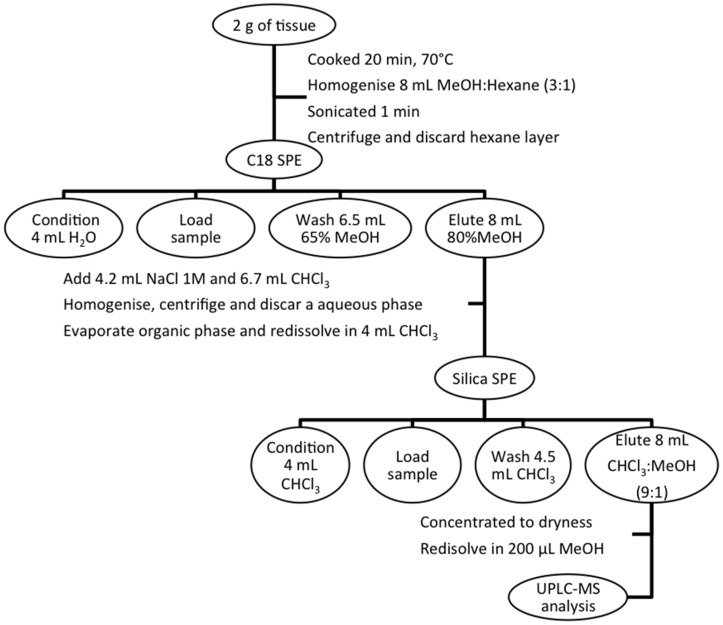
CTX purification scheme.

The Otero *et al.* (2010) extraction protocol was followed [45]. The efficiency of the method was studied by analyzing the extracts discarded in each stage of the protocol. Data showed no loss of toxin in each step. The results agree with the efficiency achieved in the method previously described (>95% for P-CTX-1B) [46]. Animals were dissected and homogenized with a blender (A320R1, 700 W, Moulinex, Lisbon, Portugal) in pooled groups in order to obtain 2 g of tissue, with the exception of *Aplysia depilans*, *Charonia lampas*, *Diadema africanum*, *Holothuria* (*Platyperona*) *sanctori*, *Marthasterias glacialis*, *Ophidiaster ophidianus*, *Paracentrotus lividus*, *Sphaerechinus granularis*, and *Umbraculum umbraculum*. In these cases, each animal was treated separately since they had enough extractable biomass. The homogenized tissue was cooked for 20 min at 70 °C, then homogenized with 8 mL of Methanol/Hexane (3:1), sonicated (1 min, 70 Hz, Vibra Cell, Sonic & Materials, Newtown, CT, USA), and subsequently centrifuged at 4000 rpm for 20 min. The upper hexane layer was discarded, and the lower methanol phase was filtered through a 0.45-µm filter (Millipore Ultrafree-MC centrifugal filter units, Bedford, MA, USA). The resulting filtered was diluted into methanol water (50:50). Thereafter Solid Phase Extraction (SPE) was performed using C_18_ SPE cartridges (500 mg/3 mL volume from Supelco, Bellefonte, PA, USA). Cartridges were previously conditioned with 4 mL of milliQ water, then samples were loaded and washed with 65% Methanol, and finally samples were eluted in 80% Methanol. Thereafter, samples were mixed with 4.2 mL of 1 M NaCl and 6.7 mL of Chloroform and centrifuged for 4 min at 2000 rpm (Centrifugal-Legend RT, Sorvall, Waltham, MA, USA). The upper methanolic layer was discarded and the lower organic layer was evaporated to dryness in a rotary evaporator (Büchi, Flawil, Switzerland) and dissolved in 4 mL chloroform. For reducing matrix interference, another cleanup procedure was done with Silica Sep-Pak cartridges (Waters, Milford, CT, USA). After loading the sample cartridges were conditioned with chloroform, samples were washed with chloroform and eluted 90% of chloroform. Extract was concentrated to dryness and then re-suspended in methanol. In Figure 5 is displayed the totality of the purification procedure. Before UPLC-MS analysis, positive samples were confirmed and the exact mass was obtained by UPLC-MS-IT-TOF.

### 3.4. UPLC-MS Conditions

For the analysis, a 1290 Infinity ultra-high-performance liquid chromatography (UHPLC) system coupled to a 6460 Triple Quadrupole mass spectrometer (both Agilent Technologies, Waldbronn, Germany) was used. Chromatographic separation was performed at 35 °C, the injection volume was 5 µL and flow rate of 0.4 mL/min using a column AQUITY UPLC BEH C18 (2.1 × 100 mm, 1.7 µm, Waters, Manchester, UK). The nitrogen generator is a Nitrocraft NCLC/MS from Air Liquid (Madrid, Spain). Mobile phases A and B were water and acetronitrile:water (95:5), respectively, both acidified with 50 mM formic acid and 2mM ammonium formate. Chromatographic separation was performed by gradient elution starting with 50% B for 2.5 min, then increasing to 100% B for 4.5 min., this condition was hold for 4.5 min and reducing afterward to 50% B over 0.1 min. This proportion was maintained for 2.4 min, until the next injection to equilibrate the system. The electrospray (ESI) source of 6460 mass spectrometer was operated with the following values of source-dependent parameters: gas temperature, 350 °C; gas flow, 8 L/min; sheath gas temperature, 400 °C; sheath gas flow, 11 L/min, nebulizer, 45 psi; capillary voltage, and 4000 V; and nozzle voltage 0 V. All analyses were performed in MS scan and selected ion monitoring (SIM).

For CTX-3C standard, a six-point calibration curve among the range 37.5-600 ng/mL was done. The limit of detection (LOD) was 1.125 µg/Kg and a limit of quantification (LOQ) was 3.75 µg/Kg.

### 3.5. UPLC-MS-IT-TOF Conditions

The UPLC system, from Shimadzu (Kyoto, Japan) consists of two pumps (LC-30AD), autoinjector (SIL-10AC) with refrigerated rack, degasser (DGU-20A), column oven (CTO-10AS) and a system controller (SCL-10Avp). The system is coupled to an IT-TOF-MS system with an electrospray ionization (ESI) interface (Shimadzu, Kyoto, Japan). The nitrogen generator is a Nitrocraft NCLC/MS from Air Liquid (Spain). The separation was performed with an ACQUITY UPLC Phenyl-Hexyl column (2.1 × 100 mm, 1.7 µm particle size, Waters, Spain). Mobile phases A and B were water and acetronitrile:water (95:5), respectively, both acidified with 50 mM formic acid and 2 mM ammonium formate. Chromatographic separation was performed by gradient elution starting with 50% B for 2.5 min, then increasing to 100% B for 4.5 min., this condition was hold for 4.5 min and reduced afterward to 50% B over 0.1 min. This proportion was maintained 2.4 min. until the next injection to equilibrate the system. The mobile phase flow rate was 0.4 mL/min, the injection volume was 5 µL and the temperature was maintained at 35 °C. The MS method was operated in positive mode with the following ESI source conditions: nebulizing gas flow, 1.5 L/min, heat block temperature and CDL temperature, 200 °C and detector voltage, and 1.65 kV. The molecules were analyzed using an ion accumulation time of 10 ms.

### 3.6. Statistical Analyses

The influence of the factors Sampling site (Morocco, Madeira and Azores islands) and organism type in the CTX occurrence was analyzed using Generalized Linear Models (GLZ). The dependent variable was the concentration of CTX in the organisms flesh (μg CTX-3C equivalents/Kg). Because only the starfish yielded positive results, primarily, we performed an analysis considering only the starfish data, using sampling site as factor. The dataset consists of CTX concentrations found in individual samples from each organism. There is one single data per pooled sample, being the pools constituted by similar numbers of organisms. The data set could be considered as a zero—inflated dataset, with variance larger than the mean. These models are usually handled with Poisson or negative binomial distributions [47]; however, we can use neither of these distribution because our data are continuous. Instead, we used the approach of gamma hurdle models [48], which performs the analysis in two steps: first, the analysis of presence/absence of the toxin (managed with a binomial or negative binomial distribution) and second, on these data showing positive concentrations of CTX, a GLZ with gamma distribution. Besides the analysis including sampling site as factor, we performed another analysis considering starfish species as factor.

All the models were performed with R software [49], package stats (), function glm.

## 4. Conclusions

The primary aim of this work was to search for new vectors for CTX in the Portuguese islands and the northwestern coast of Morocco using UPLC-IT-TOF-MS and UPLC-MS/MS techniques. From 22 surveyed species, we detected CTX in two species of starfish: *M. glacialis* and *O. ophidianus*. In addition, the quantifiable results were all in Portuguese territory, being São Miguel Island (Azores), the location with greater propensity to find these biotoxin groups. This is an important finding since it is, to the best of our knowledge, the first report of these toxins in echinoderms ever. We detected three analogues, CTX-1B and two unnamed derivatives, in concentrations that ranged from 4.40 to 124.04 μg CTX-3C equivalents/Kg fw, especially in the red starfish, *O. ophidianus*. Since this species has detritivorous feeding habits, this could be the main explanation for considerable difference with regard to accumulated amounts, comparing to the predatory spiny starfish (*M. glacialis*). Regarding Ciguatera monitoring, starfish present themselves as a good alternative, though more studies should be done in order to understand correlation of CTX uptake between echinoderms and predatory fish, to better evaluate human health risk.
